# Evaluation of the immunomodulatory effects of a South African commercial traditional immune booster in human peripheral blood mononuclear cells

**DOI:** 10.1186/s12906-016-1294-7

**Published:** 2016-08-22

**Authors:** Mlungisi Ngcobo, Nceba Gqaleni

**Affiliations:** 1 Traditional Medicine Laboratory, School of Nursing and Public Health, College of Health Sciences, University of KwaZulu Natal, Durban, South Africa; 2 Department of Public Management and Economics, Faculty of Management Sciences, Durban University of Technology, Durban, South Africa

**Keywords:** Cytokines, Cytotoxicity, Immune booster, Soluble receptors, Traditional medicines

## Abstract

**Background:**

With the burden of HIV and AIDS still very high, South Africa has seen an increase in commercial traditional medicines claiming to have immune-enhancing effects. Because of lack of regulation of the traditional medicine sector, these products have proliferated. This study aimed to evaluate the immunomodulatory effects of *uMakhonya®*, a commercial traditional immune booster, using various models of normal human peripheral blood mononuclear cells (PBMCs).

**Methods:**

Immunosuppressed, mitogen-, and peptidoglycan (PG)-stimulated PBMCs were treated with various doses of *uMakhonya®* and incubated for 24 h. The treated and control samples were analyzed for cytotoxicity, secretion of 12 different inflammatory cytokines, soluble interleukin-2 receptor (sIL-2R) levels, and nitric oxide (NO) secretion.

**Results:**

In cytotoxicity assays, *uMakhonya®* induced dose-dependent cytotoxic effects in all three models, with IC_50_ values of 512.08, 500, and 487.91 μg/mL for immunosuppressed, phytohaemagglutinin (PHA)-, and PG from *Staphylococcus. aureus* (PG-*S. aureus*)-stimulated PBMCs, respectively. *UMakhonya®* at 100 and 10 μg/mL induced a significant (*p* < 0.05) increase in the secretion of IL-1α, IL-1β, IL-6, IL-10, tumor necrosis factor alpha (TNF)-α, and granulocyte-macrophage colony-stimulating factor (GM-CSF) in cyclosporine-, immunosuppressed, and PHA-stimulated PBMCs. In the same samples, there was a significant increase (*p* < 0.05) in sIL-2R concentration, which correlated with an increase in the secretion of inflammatory cytokines. In PBMCs stimulated with PG-*S. aureus*, *uMakhonya®* at doses of 100 and 10 μg/mL significantly (*p* < 0.05) suppressed the secretion of inflammatory cytokines, especially IL-1β and TNF-α. PG-*S. aureus-*stimulated PBMCs also showed a significant decrease (*p* < 0.05) in sIL-2R concentration when compared to control samples. *UMakhonya®* insignificantly (*p* > 0.05) decreased NO levels in PBMCs after PG-*S. aureus* stimulation.

**Conclusions:**

These results showed that *uMakhonya®* can induce both pro-inflammatory and anti-inflammatory effects depending on the initial stimuli applied to immune cells.

## Background

Modernization and the technology age have brought about a change in the operation of African traditional medicine (ATM) and the practice of traditional healing. Suppliers of traditional medicine include retail *muthi* (herbal) shops, pharmacies, pharmaceutical manufacturers, herbal hawkers, and herbal wholesalers [1]. Many of the traditional medicine manufacturers do not conform to Good Manufacturing Practice (GMP) standards [2]. Commercial products based on traditional knowledge have not only tapped the traditional theories of tonics but also have other added claims such as immune enhancing properties and antimicrobial activities defined as per the Western biomedical concepts. The HIV/AIDS epidemic has also fueled the demand, with many patients using the so-called immune boosters that are sold commercially [3, 4]. The increased use of herbal plants and traditional medicine formulations necessitates development of a mechanism for rapid evaluation of the medicinal applications of these products.

Plant-derived immune tonics are widely used as a supplement for maintenance of health. African tonic plants are used to reduce fatigue, improve general health (during or after illness), lower stress, and cleanse the blood [5]. Based on their reported immunomodulatory effects, plant-derived compounds have emerged as prime therapeutic options in recent times, leading to their rigorous scientific examination to determine efficacy and safety [6]. Immunomodulation is a very complex theme, related to many diseases, with many targets for drugs, and therefore may present with many modes of action [7]. Modulation of the immune system denotes any change in the immune response; this may involve induction, expression, amplification, or inhibition of any phase or part of that response [8]. Complex mixtures of traditional medicinal herbs thought to have immunomodulatory effects are a fixture of ATM and may include several compounds that exhibit activity against different targets or may even have synergistic effects [7]. Numerous in vitro methods have been developed to evaluate the modes of action of these potential immunomodulators [9]. Selection of proper immune cells from suitable immune organs allows for the understanding of the mode of action of immunomodulation [10].

Novel cell receptors and signal transduction molecules produced by immune cells have been identified and these regulate the host response to invading microorganisms. Research has been focused on finding compounds and substances that can modulate the biologic immune response and enhance the host’s ability to resist disease [11]. In this study, we aimed to evaluate the immunomodulatory effects of *uMakhonya®*, a widely used herbal immune booster in South Africa, by using various models of human PBMCs. In an earlier study, this traditional medicine was shown at non-cytotoxic doses is not a chemoattractant but is able to significantly induce chemokine secretion in LPS stimulated and unstimulated THP-1 monocytes. The increase in chemokines secretion may involve the activation of the NF-қβ signal transduction pathway [12]. We developed three different models of PBMCs representing normal replication (PHA stimulation), immunosuppression (cyclosporine-treated), and infection (PG-*S. aureus* stimulation) of immune cells in order to analyze *uMakhonya®* cytotoxicity and its effects on inflammatory cytokines, receptor levels, and nitric oxide (NO) secretion. The interest in such a product and in the thousand other similar commercial products is made relevant by their use by patients with a variety of ailments, most of them related to HIV infection and AIDS. These products, based on traditional knowledge, have become synonymous with boosting the immune system to fight opportunistic infections. [4].

## Methods

### Preparation of *uMakhonya*® formula

*UMakhonya***®** formulation samples (Batch number: R0101, National Pharmaceutical Product Index (NAPPI) Code: 710345–001) were a gift from the owner of the traditional medicine formulation, Mr. Victor Ndlovu. This product is formulated by combining five different plant extracts. The herbal plants are listed on the packaging of the formula sold in supermarkets, and each 5 mL of *uMakhonya***®** contains 90 mg *Artemisia afra* (African wormwood, *Compositae* family), 8.5 mg menthol (produced by *Lamiaceae* family, mostly those of the *Mentha* genus), 0.3 mL *Psidium guajava* liquid extract (guava, *Myrtaceae* family), 0.08 mL *Chondrus crispus* (Irish moss, *Rhodophyceae* family), and 0.06 mL *Uncaria tomentosa* (cats-claw tincture, *Rubiaceae* family), all extracted in water. Chemical fingerprints of *uMakhonya***®** using gas chromatography–mass spectrometry (GC-MS) and nuclear magnetic resonance (NMR) are provided as additional data. The formulation is listed with the Medicines Control Council (MCC) of South Africa and exclusive rights are reserved. It was manufactured by UMakhonya Natural Health Products (Pty) Ltd (Pinetown, South Africa). At the manufacturing phase, the plants were mixed proportionally in a 25-l tank and then extracted using tap water by boiling the tank overnight. The extract was then cooled, filtered once with steel sift followed by removal of finer particles using a sifting net, and then packaged into 1- and 5-l containers.

To prepare the extract for in vitro studies, the liquid extract was further sterile-filtered and then freeze-dried to powder. The powdered plant material was then reconstituted at 10 mg/mL in phosphate-buffered saline (PBS) and this was further sterile-filtered using 0.22 μm filters. This stock solution was then serial diluted to working concentrations of 1000, 500, 100, 50, and 10 μg/mL using complete culture media (CCM). Endotoxin contamination was measured using the Limulus Amebocyte Lysate (LAL) QVL-1000^TM^ (Lonza, USA) with a sensitivity of 0.1 endotoxin units (EU) per mL (supplementary data, Table 1). Polymyxin B sulfate (10 μg/mL) was added to reduce the immunostimulatory effects due to endotoxin contamination. The recommended dose of *uMakhonya***®** according to the packaging is about 150 mL per day of the liquid concentrate. This volume equated to about 800 mg of freeze dried material [12].

**Table 1:** Measurement of nitrite radicals as a measure of the levels of nitric oxide secretion in PBMCs stimulated with PG-*S. aureus* (100 μg/mL) and treated with various doses of *uMakhonya®*. Treatment of stimulated PBMCs with various doses of *uMakhonya®* did not effect a significant increase (*p* > 0.05) in NO secretion when compared to untreated, PG-*S. aureus*-stimulated-only and a significant increase (*p* > 0.05) in NO secretion when compared to PG-stimulated plus cyclosporine-treated samples

	Untreated PBMCs	PG-*S. aureus*	PG-*S. aureus* plus Cyclosporine	*PG-S. aureus* plus *uMakhonya®* (μg/mL)		
NO concentration (μM)				100	50	10
1	9.21	10.12	10.24	9.15	9.33	9.03
2	9.21	9.82	10	9.15	9.56	9.27
3	10.55	11.52	11.33	10.48	10.79	11.03
Mean ± SEM	9.66 ± 0.770	10.49 ± 0.905	10.53 ± 0.710	9.60 ± 0.770	9.90 ± 0.780	9.78 ± 1.092

*SEM* standard error of the mean

### Cell culture

Normal human whole blood was carefully layered onto equal amounts of Histopaque 1077 and then centrifuged at 600 *g* for 30 min at 25 °C. After centrifugation, the buffy coat layer containing PBMCs was isolated and washed twice in 5 mL PBS (300 *g* for 20 min at 25 °C). The final pellets were re-suspended in complete culture media at 1 × 10^6^ cells/mL and then different models of PBMCs were prepared according to Leung et al., [13] with some variations. Briefly, the PBMCs were stimulated with either PHA/PG-*S. aureus* or immunosuppressed with 20 μg/mL of cyclosporine for 2 h. Without eliminating the stimulation or immunosuppressive effect of cyclosporine, the cells were aliquoted to 6-well plates and treated with *uMakhonya®* at doses ranging from 1000 μg/mL to 10 μg/mL at a ratio of 1:1. The treated PBMCs were then incubated for 24 h at 37 °C, 5 % CO_2_ and 95 % humidity. At the end of the incubation period, the cells and their supernatants were used for further experiments.

### Cell viability assay

The luminescent cell viability ATP assay kit from Promega uses recombinant luciferase to catalyze the following reaction:

ATP + d-Luciferan + O_2_ → Oxyluciferan + AMP + PPi + CO_2_ + Light (560 nm).

When ATP is the limiting component in the reaction, the intensity of the emitted light is proportional to the concentration of ATP. Based on these principles, the levels of ATP in PBMCs stimulated with either PHA/PG-*S. aureus* or immunosuppressed with 20 μg/mL of cyclosporine and treated with doses of *uMakhonya***®** were analyzed according to manufacturer instructions. Cyclosporine (20 μg/mL) was used as a positive control for cytotoxicity against immune cells. Briefly, a sample (100 μL) of 24 h treated/control cell suspension was pipetted into three different wells of a white opaque 96-well plate. The working CellTiter-Glo^TM^ Reagent (cat number: G7570) was prepared immediately before use and was added to the wells with treated cells at 100 μL per well. The plate was agitated on a plate shaker for 2 min at 150 *g* and incubated in darkness for 10 min at room temperature. At the end of the incubation period, the plate was loaded into the luminometer and the relative light units (RLU) of the samples were measured. Background signals of cell culture media and *uMakhonya***®** doses (negative control) were subtracted from each average read. A dose response curve was also generated for the ATP levels using RLU versus different concentrations of samples. The cell viability assay was done in triplicate and repeated three times before the follow-up assays were undertaken.

### Cytokine secretion assay

The Multi-Analyte Profiler ELISArray assay kit (Qiagen, USA) is designed to be used with supernatants from treated cells or with serum from whole blood. For the purpose of this research, inflammatory cytokines were analyzed from supernatants of treated models of PBMCs and control samples. Each kit included a 96-well plate coated with antibodies for the various chemokines in the microarray. Each row of the plate from 1 to 12 represented a single cytokine in the following order: IL-1α, IL-1β, IL-2, IL-4, IL-6, IL-8, IL-10, IL-12, IL-17α, interferon gamma (IFN-γ), tumor necrosis factor alpha (TNF-α), and granulocyte-macrophage colony-stimulating factor (GM-CSF). The kit had negative and positive controls. Each sample was assayed in duplicate.

The ELISA analyses were performed according to manufacturer instructions. Briefly, incubation of the samples in the 96-well plates allowed the captured antibodies to bind to their specific protein of interest. Cyclosporine-treated, PHA-, and PG-*S. aureus*-stimulated samples treated with *uMakhonya®* (100 and 10 μg/mL) and untreated control samples were analyzed. After removing the unbound protein with wash buffer, biotinylated detection antibodies (50 μL) were added to the wells to bind to the captured analyte. Following another wash, an avidin-horseradish peroxidase conjugate (100 μL) was added. The wells were again washed and the colorimetric substrate solution was added, developing to a blue color in direct proportion to the amount of protein analyte present in the initial sample. The color development was stopped by adding the stop solution, and the absorbance was read at 450 nm with reference at 570 nm in a microplate reader as per manufacturer instructions. Secretion of cytokines was measured in duplicate and two independent experiments were done.

### IL-2 receptor ELISA

Analyses of human soluble IL-2 receptor (sIL-2R) levels were carried out using the IL-2 Receptor Human ELISA Kit from ABCAM® (England) and done according to the provided protocol. Briefly, samples (100 μL each) of supernatants from PBMCs treated with different doses of *uMakhonya®* plus standards of known IL-2 receptor concentrations and control samples were pipetted into separate wells and incubated with biotinylated monoclonal antibodies specific for sIL-2R (50 μL) for 3 h at room temperature. This was followed by the addition of streptavidin peroxidase enzyme (100 μL). After incubation for 30 min and washing to remove the unbound enzyme, a substrate solution (100 μL) was added, which produced a colored reaction. The intensity of this colored product is directly proportional to the concentration of sIL-2R present in the samples. The enzyme-substrate reaction was stopped by quickly pipetting 100 μL of sulfuric acid. The results were read immediately on a Zenyth200 spectrophotometer at 450 nm as the primary wavelength and 610 nm as the reference wavelength. The experiments were done in triplicate and repeated twice per dose.

### Nitric oxide secretion

The Griess reagent system from Promega (USA) was used to measure nitrite (NO_2_^−^), which is one of two primary, stable, and nonvolatile breakdown products of NO. To perform the assay, 50 μL of supernatants from peptidoglycan-stimulated and control PBMCs were plated in triplicate on 96-well plates. The samples were left to equilibrate to room temperature after which 50 μL of Sulfanilamide Solution was dispensed to all experimental samples and incubated for 10 min at room temperature away from light. Then, 50 μL of *N*-1-napthylethylenediamine dihydrochloride (NED) solution was dispensed to all sample wells and the plate incubated for another 10 min at room temperature, again protected from light. Absorbance was then measured within 30 min on a Zenyth200 plate reader at 540 nm. Nitrite standards were included as part of the samples and were used to draw a reference curve. All samples and standards were done in triplicate and the experiments were repeated twice.

### Statistical analysis

Data analyses were done on Microsoft Excel to obtain descriptive statistics. The IC_50_ for cytotoxicity and different levels of significance within the different treated groups were analyzed using one-way analysis of variance (*ANOVA*) and the differences between the treated cells, the untreated cells, and the negative control samples were analyzed using GraphPad Prism (version 5) software with the *Tukey-Kramer* multiple comparison test. Differences with *p* ≤ 0.05 were considered statistically significant.

## Results

### Cell viability

**Fig. 1:**
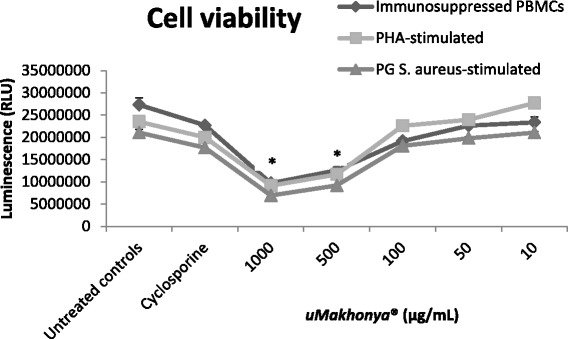
The cytotoxic of effects of *uMakhonya®* on three different models of PBMCs, immunosuppressed, PHA-stimulated, and *S. aureus* peptidoglycan (PG-*S. aureus*)-stimulated cells. In all three models, *uMakhonya®* induced significant (*p* < 0.05) cytotoxicity at high doses (1000 to 500 μg/mL) while the effect of lower doses (100 to 10 μg/mL) was not significant (*p* > 0.05). The IC_50_ values of 512.08, 500, and 487. 91 μg/mL were recorded for immunosuppressed, PHA-stimulated, and PG-*S. aureus*-stimulated cells, respectively. Further experiments were performed with lower doses ranging from 100 to 10 μg/mL, which did not show significant cytotoxicity

In all three models (cyclosporine-suppressed, PHA-stimulated, and PG-*S. aureus*-stimulated PBMCs), *uMakhonya®* induced significant (*p* < 0.05) cytotoxicity at high doses (1000 to 500 μg/mL) while the effect of lower doses (100 to 10 μg/mL) was not significant (*p* > 0.05). The cytotoxicity of the high doses of *uMakhonya®* was significantly greater (*p* < 0.05) than that of the immunosuppressive drug cyclosporine (Fig. 1). The IC_50_ values of 512.08, 500, and 487. 91 μg/mL were recorded for immunosuppressed, PHA-stimulated, and PG-*S. aureus*-stimulated PBMCs, respectively. Further experiments were performed with lower doses of *uMakhonya®* ranging from 100 to 10 μg/mL, which did not show significant cytotoxicity.

### Cytokine secretion assay

**Fig. 2:**
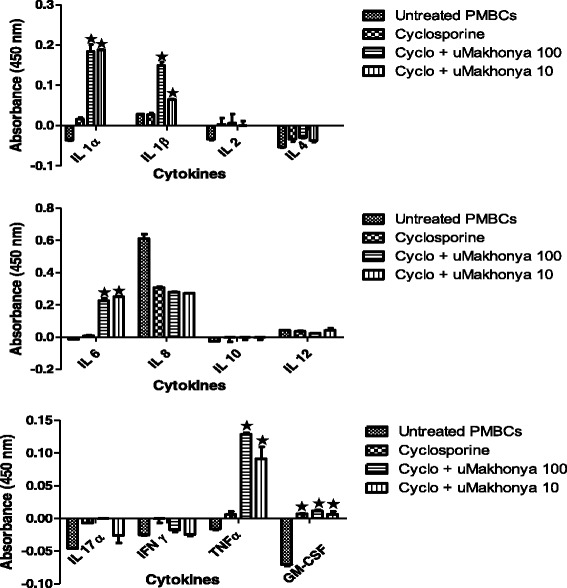
Effects of *uMakhonya®* at doses of 100 and 10 μg/mL on the secretion of 12 inflammatory cytokines over 24 h in PBMCs pre-treated with cyclosporine (20 μg/mL) for 2 h. *UMakhonya®* significantly increased (*p* < 0.05) the secretion of IL-1α, IL-1β, IL-6, and TNFα when compared with cyclosporine-only treatment. Secretion of IL-2, which is the target of suppression by cyclosporine, did not significantly increase after the addition of *uMakhonya®*

In the immunosuppressive model, the effects of *uMakhonya®* doses (100 and 10 μg/mL) on the secretion of 12 inflammatory cytokines over 24 h in PBMCs pre-treated with cyclosporine (20 μg/mL) for 2 h were analyzed. *uMakhonya®* significantly increased (*p* < 0.05) the secretion of IL-1α, IL-1β, IL-6, and TNFα compared with cyclosporine-only treatment. Secretion of IL-2, which is the target of suppression by cyclosporine, did not significantly increase after the addition of *uMakhonya®* (Fig. 2). Overall, the addition of *uMakhonya®* stimulated the secretion of inflammatory cytokines.

**Fig. 3:**
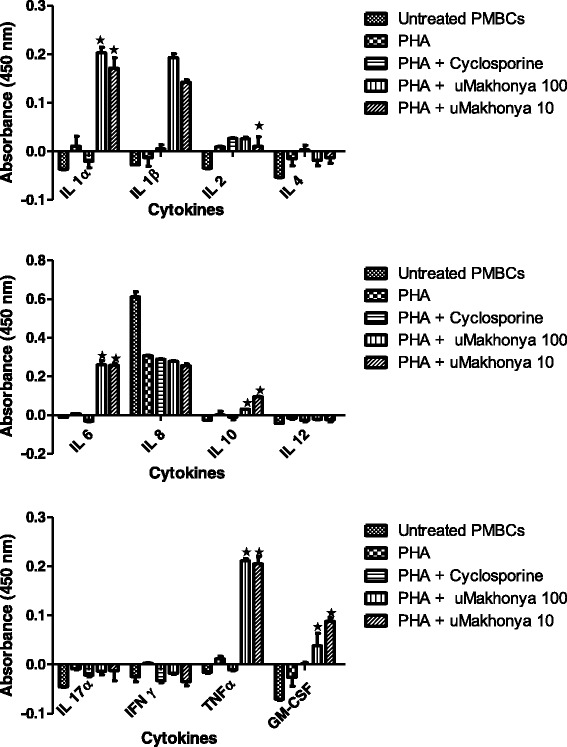
Immunomodulatory effects of *uMakhonya®* at doses of 100 and 10 μg/mL on the secretion of 12 inflammatory cytokines over 24 h in PBMCs pre-treated with PHA (20 μg/mL) for 2 h. *UMakhonya®* significantly increased (*p* < 0.05) the secretion of IL-1α, IL-1β, IL-6, IL-10, TNFα, and GM-CSF when compared cyclosporine-only treatment. IL-2 secretion was not significantly affected (*p* > 0.05) by the addition of *uMakhonya®* compared to that observed for PHA plus cyclosporine treatment

In the mitogen-stimulated model, the immunomodulatory effects of *uMakhonya®* were analyzed at doses 100 and 10 μg/mL on the secretion of 12 inflammatory cytokines over 24 h in PBMCs pre-treated with PHA (20 μg/mL) for 2 h. *UMakhonya®* significantly increased (*p* < 0.05) the secretion of IL-1α, IL-1β, IL-6, IL-10, TNFα, and GM-CSF when compared to cyclosporine-only treatment. IL-2 secretion was not significantly affected (*p* > 0.05) by the addition of *uMakhonya®* compared to that observed after PHA plus cyclosporine treatment (Fig. 3). Similar to the immunosuppressive model, the mitogen-stimulated model exhibited increased secretion of inflammatory cytokines after the addition of *uMakhonya®*.

**Fig. 4:**
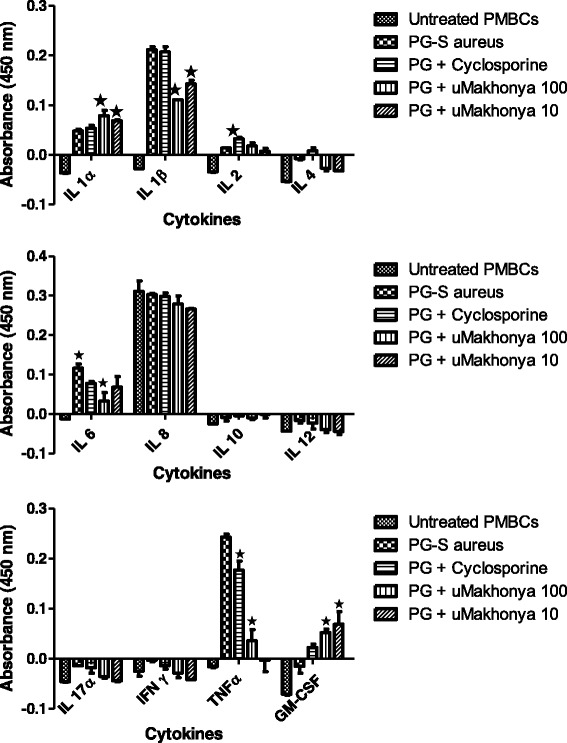
Immunomodulatory effects of *uMakhonya®* at doses of 100 and 10 μg/mL on the secretion of 12 inflammatory cytokines over 24 h in PBMCs pre-stimulated with PG-*S. aureus* (100 μg/mL) for 2 h. Secretion of IL-1β, IL-4, IL-6, IL-12, IL-17α, IFNγ, and TNFα significantly decreased (*p* < 0.05) after the addition of *uMakhonya®* compared to that in PG-*S. aureus*-treated samples. Secretion of IL-2 did not show a significant change when compared to control samples. Secretion of only IL-1α and GM-CSF was increased (*p* < 0.05) by the addition of *uMakhonya®* to samples stimulated with PG-*S. aureus*

In the infection model, the immunomodulatory effects of *uMakhonya®* were analyzed at doses of 100 and 10 μg/mL on the secretion of 12 inflammatory cytokines over 24 h in PBMCs pre-stimulated with PG-*S. aureus* (100 μg/mL) for 2 h. Secretion of IL-1β, IL-4, IL-6, IL-12, IL-17α, IFNγ, and TNFα significantly decreased (*p* < 0.05) after the addition of *uMakhonya®* compared to that in PG-*S. aureus*-treated samples. IL-2 secretion was not significantly affected when compared to that in control samples. Secretion of only IL-1α and GM-CSF increased (*p* < 0.05) after the addition of *uMakhonya®* to samples stimulated with PG-*S. aureus* (Fig. 4). Unlike the immunosuppressive and mitogen models, overall the addition of *uMakhonya®* suppressed the immunostimulatory effects of PG-*S. aureus* on the secretion of inflammatory cytokines.

### IL-2 receptor ELISA

**Fig. 5:**
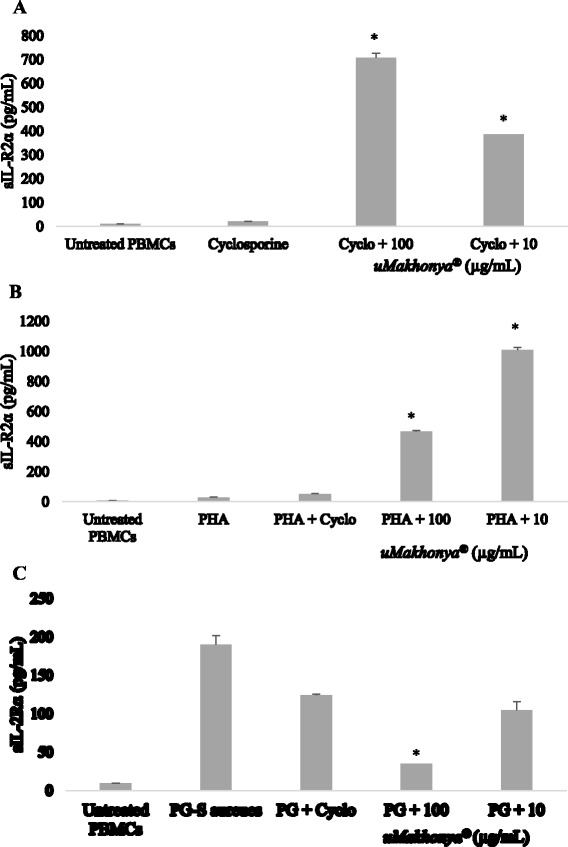
Soluble IL-2 receptor levels in supernatants of cyclosporine-immunosuppressed (**A**), PHA-stimulated (**B**), and PG-*S. aureus*-stimulated (**C**) PBMCs treated with *uMakhonya®* doses (100 and 10 μg/mL) over 24 h. In immunosuppressed and PHA-stimulated samples, addition of *uMakhonya®* significantly increased (*p* < 0.05) sIL-2R levels compared to untreated and cyclosporine-suppressed samples. In PG-*S. aureus*-stimulated samples, the addition of *uMakhonya®* caused a significant decrease (*p* < 0.05) in sIL-2R level when compared to samples stimulated with PG-*S. aureus* only. The highest dose of *uMakhonya®* (100 μg/mL) was more effective (*p* <0.05) at reducing sIL-2R in PG-*S. aureus*-stimulated samples than cyclosporine

Soluble IL-2 receptor levels were analyzed in order to find out whether their levels correlated with the secretion of inflammatory cytokines in the three different PBMC models. In immunosuppressed and PHA-stimulated samples, the addition of *uMakhonya®* significantly increased (*p* < 0.05) sIL-2R levels when compared to untreated and cyclosporine-suppressed samples (Fig. 5a-b). This correlated with the overall pro-inflammatory effects of *uMakhonya®* on cytokine secretion. In PG-*S. aureus*-stimulated samples, the addition of *uMakhonya®* caused a significant decrease (*p* < 0.05) in sIL-2R level when compared to samples stimulated with PG-*S. aureus* only. The highest dose of *uMakhonya®* (100 μg/mL) was more effective (*p* < 0.05) at reducing sIL-2R in PG-*S. aureus*-stimulated samples than cyclosporine (Fig. 5c). The decrease in sIL-2R levels in PG-*S. aureus*-stimulated samples also correlated with the overall anti-inflammatory effects of *uMakhonya®* on cytokine secretion in the same model.

### Nitric oxide secretion

Treatment of PG-*S. aureus*-stimulated PBMCs with various doses of *uMakhonya®* did not effect a significant change (*p* > 0.05) in NO secretion when compared to untreated, PG-*S. aureus*-stimulated only and PG-*S. aureus*-stimulated plus cyclosporine-treated samples. While stimulation of PBMCs with PG-*S. aureus* caused a slight increase in NO secretion when compared to unstimulated cells, the addition of *uMakhonya®* caused a non-significant decrease in secretion (Table 1).

## Discussion

In this study, we investigated the in vitro immunomodulatory effects of *uMakhonya®*, a widely sold South African immune booster product based on ATM knowledge. *uMakhonya***®** immune booster, according to the product labeling, is indicated to improve one’s immune system. It also claims to cleanse the blood and to control diabetes, high blood pressure, pneumonia, shingles, ulcers, and hemorrhoids. The results of this study showed that *uMakhonya®* is significantly cytotoxic to PBMCs at high doses (Fig. 1) with IC_50_ values of 512.08, 500, and 487. 91 μg/mL recorded for immunosuppressed, PHA-stimulated, and PG-*S. aureus*-stimulated PBMCs, respectively. This is indicative of in vivo toxicity at excessively higher equivalent doses. The recommended dose of *uMakhonya***®** according to the packaging is about 150 mL divided into three separate doses per day. This volume equated to about 800 mg of freeze dried material. Although it would be difficult to extrapolate the in vitro results to a full human physiology, this recommended dosage seems minimal to cause any adverse toxic effects. *uMakhonya®* at non-cytotoxic doses ranging from 100 to 10 μg/mL were shown to stimulate the secretion of pro-inflammatory cytokines and increase sIL-2R levels in immunosuppressed and PHA-treated PBMCs (Figs. 2 and 3). However, this product showed anti-inflammatory effects by significantly decreasing inflammatory cytokines and sIL-2R levels in PG-*S. aureus*-stimulated PBMCs (Figs. 4 and 5c). Many advances have been made recently in the understanding of the functioning of the immune system. Whether immunomodulators enhance or suppress the immune response can depend on a number of factors, including dosage as well as route and timing of administration. This response can also depend on the mechanism of action or site of activity [11]. Therefore, in choosing research models to study immunomodulators, a careful and precise process must be followed in order to ensure that the results of such research reflect the absolute nature and mechanisms of action of the compounds in question. *uMakhonya®* demonstrated that its mode of action depends on the type of stimuli applied to an in vitro immune system and that these effects are dose-dependent.

Cytokines are redundant secreted proteins with growth, differentiation, and activation functions that regulate and determine the nature of the immune responses [14]. Therefore, altering cytokine expression and targeting their receptors may offer therapeutic potential [15]. The addition of *uMakhonya®* antagonized the immunosuppressive effects of cyclosporine and potentiated the cell proliferation effects of PHA by increasing the secretion of pro-inflammatory cytokines including IL-1α, IL-1β, IL-6, IL-10, TNFα, and GM-CSF. PHA, a lectin isolated from red kidney bean (*Phaseolus vulgaris*), primarily stimulates T cell proliferation and it has a slight effect on B cells. This lectin stimulate blastogenesis of T lymphocytes by interaction with CD2 to stimulate the production of IL-2 and IFN-γ [10]. For this study it was observed through repeated stimulation of PBMCs that proliferative effect of PHA normally peaks after 48 h of stimulation. But the design of the studies necessitated that the stimulation be standardised along with that of immunosuppression with cyclosporine and stimulation with peptidoglycan. Therefore the stimulatory effects of PHA on cytokines are not reflected in the inflammatory cytokines secretion but the sIL-2R assay clearly demonstrates the stimulatory effects of PHA. Immunostimulants are envisaged to enhance the body’s resistance to infection by acting through both the innate and adaptive immune responses [9]. There was a significant correlation between the increase in pro-inflammatory cytokine secretion and the increase in sIL-2R concentration in both immunocompromised and mitogen-stimulated PBMCs treated with *uMakhonya®*. In vitro cellular activation has been observed to lead to not only the synthesis of cell-associated IL-2R but also a soluble form of the receptor, which could be found in cell-free supernatants. The generation of sIL-2R is not a result of cell death and the rate of release of this molecule is in proportion to its cell surface expression [16]. The association between the increased levels of sIL-2R and immunostimulation has been demonstrated in various inflammatory diseases in vivo. Siedler et al., [17] showed that patients with chronic liver disease (CLD) had significantly elevated serum sIL-2R levels compared with controls and this was associated with pro-inflammatory cytokines including IL-2, IFNγ, or IL-6, and chemokines. The shedding of sIL-2R may also limit the responsiveness of immune cells to IL-2 stimuli, thereby averting any risk of an excessive immune response which can lead to inflammatory disorders [18, 19]. In healthy individuals, this immunostimulation may serve as a prophylactic, while in immunocompromised individuals, it may act as an immunotherapeutic [9]. Immunomodulation using traditional medicinal plants can be used as an alternative to, or in conjunction with, conventional therapy for a variety of diseases, especially when host defenses have to be activated under the conditions of impaired immune response [20]. It was also determined that this traditional medicine had non-significant levels of endotoxin contamination at 1.8 endotoxin units (EU/mL) (additional files, Table 1). The endotoxin levels were removed by addition of 10 μg/mL of polymyxin B sulphate to ensure that the assessed immune effects are directly as a result of *uMakhonya***®**.

Pre-treatment of PBMCs with PG-*S. aureus* significantly increased the secretion of pro-inflammatory cytokines (Fig. 4). Peptidoglycan is a component of both gram-positive and gram-negative bacterial cell walls and is a functional lipopolysaccharide (LPS) analog. It therefore possesses potent pro-inflammatory properties in vitro including the induction of IL-1 and TNFα [21]. The addition of *uMakhonya®* to the stimulated PBMCs significantly decreased the concentration of pro-inflammatory cytokines, especially IL-1β and TNFα. The anti-inflammatory effects of *uMakhonya®* correlated with the significant decrease in sIL-2R levels in supernatants of PG-*S. aureus*-stimulated PBMCs (Fig. 5c). These anti-inflammatory effects are in contrast to its intended use, which is aimed at stimulating the immune system in response to infections. Alternatively, these anti-inflammatory effects may serve to control the immune response during bacterial infections because during infection, peptidoglycan recognition drives both cell-autonomous and whole organism defense responses [22]. In vitro studies have shown that the overall pharmacological and therapeutic effects of medicinal plants often do not derive from a single compound but from several compounds generating synergistic activity [15]. In this study, we have demonstrated that this extends to formulations of multiple medicinal plants. Such findings have led some researchers to propose that multi-component pharmacological agents that target multiple sites affect the complex physiological response more favorably than drugs that act on a single target [15]. Herbalists have known for centuries the benefit of using a combination of herbal remedies such as *uMakhonya®*, (single extracts from medicinal plants and combined extracts of different plants) in switching on the body’s defense mechanisms and self-healing and protective processes [23].

There are limited studies on the immune effects of the five medicinal plants that make up *uMakhonya®* but the available data show that the majority of these medicinal plants possess anti-inflammatory effects. *Chondrus crispus* is indigenous to the Northern Hemisphere and is the only medicinal plant extract known to induce an inflammatory response in vitro and in animal models. Seaweeds are not only rich in polysaccharides, minerals, and certain vitamins but they also contain bioactive substances such as proteins, lipids, and polyphenols. These bioactives are known to possess potent antibacterial, antiviral, and antifungal properties [24–26]. *C. crispus* has antiviral properties and is said to be particularly useful for dislodging mucus [27]. *C. crispus* and its resultant component from the extract, carrageenan, are known to be potent inflammatory agents in rodents and stimulate mice immune cells to produce TNFα in response to bacterial LPS [28, 29]. *Artemisia afra* is used traditionally to treat respiratory ailments and fever in combination with *Lippia javanica*, suggesting its immune enhancing potential. Decoctions of *A. afra* are taken as blood purifiers for acne and boils. This combined with the apparent ability to reduce stress may confer upon *A. afra* the status of an immune tonic [5, 30]. *Uncaria tomentosa* (cat’s claw) is a medicinal plant from the Amazon forest in South America with a long tradition of use in the treatment of a wide range of diseases including arthritis, gastritis, osteoarthritis, diabetes, and cancer [31, 32]. A study on human volunteers given *Uncaria tomentosa* at 350 mg daily caused a small increase in their white blood cell counts. A follow-up clinical trial where 250–350 mg of formulated *U. tomentosa* was given increased leukocyte count over six weeks in healthy volunteers [33]. Cat’s claw is a remarkably potent inhibitor of TNFα production. The primary mechanism appears to be immunomodulation via suppression of TNFα synthesis [34]. *Psidium guajava* (guava) is an important food crop and medicinal plant in tropical and subtropical countries. Different parts of this plant are used widely in traditional medicine practices worldwide [35, 36]*.* Many pharmacological studies have demonstrated the antioxidant, hepatoprotective, anti-allergic, antimicrobial, antigenotoxic, antiplasmodial, cytotoxic, antispasmodic, cardioactive, antitussive, antidiabetic, anti-inflammatory, and anti-nociceptive activities of guava, supporting its traditional uses [37–39]. In addition to tobacco, menthol is used in a variety of products such as foods, topical therapeutic preparations, and oral hygiene and dental formulations [40]. Menthol has been shown to have analgesic and related anti-inflammatory activities by its effect on transient receptor potential cation channel subfamily M member 8 (TRPM8), Kappa receptor stimulation, and inhibition of voltage gate sodium channels [41]. The complexity of active compounds produced by the medicinal plants constituting *uMakhonya®* may therefore explain the differences in response in the three PBMCs models used in this study.

Peptidoglycan is also a potent activator of inducible nitric oxide synthase (iNOS) as part of its in vitro pro-inflammatory effects. Activation of the iNOS by PG leads to increased production of NO [42, 43]. The measure of nitrite as a reflection of NO concentration in PG-*S. aureus*-stimulated PBMCs showed that treatment with *uMakhonya®* caused a non-significant reduction in NO secretion when compared to other stimulated cell samples (Table 1). In terms of the concentration of NO secreted by PBMCs, Jacob et al. [44] showed that the amount of NO released from unstimulated PBMCs cultured from control blood samples measured using the Griess reagent system in the culture supernatants was 11.27 ± 2.5 μM and 8.91 ± 1.5 μM NO at 24 h and 48 h, respectively. These values are comparable with the results obtained in this study. NO is a physiological mediator produced by many cells involved in immunity and inflammation. NO is subsequently oxidized to nitrite and nitrate, reactive nitrogen species (RNS) that mediate most of its immunological effects [45]. Therefore the reduction of nitrite by *uMakhonya®* may reflect its antioxidant effects. The oxidant scavenging properties may also explain the anti-inflammatory effects of *uMakhonya®* in PG-*S. aureus*-stimulated PBMCs. The five herbal medicines constituting *uMakhonya®* have all been shown to possess potent antioxidant properties in separate experiments [33, 46–49]. Immunostimulants, as *uMakhonya®* is purported to be, can be used both by healthy individuals and persons with impaired immune systems. In healthy individuals they are expected to act as prophylactic agents and in individuals with impairment of the immune response as immunotherapeutic agents [9]. A study on the immunomodulatory effects of goldenseal and *Astragalus* extracts showed that herbal medicines may function to moderate symptoms associated with overactive pro-inflammatory responses, with each having a distinct subset of modulatory effects on cultured macrophages [50]. This may provide a possible explanation for the observed pro- and anti-inflammatory effects of by *uMakhonya®* doses under different physiological stimuli. The potential uses of immunomodulators in clinical medicine include treatment of immunodeficiency caused by AIDS and suppression of immune response in autoimmune disease [10].

## Conclusions

This study showed that *uMakhonya®* is significantly cytotoxic to PBMCs at high doses. *uMakhonya®* was also shown to stimulate the secretion of pro-inflammatory cytokines and to increase sIL-2R levels in PBMCs immunosuppressed with cyclosporine or mitogen-stimulated with PHA. In PG-*S. aureus*-stimulated PBMCs, *uMakhonya®* showed anti-inflammatory effects by suppressing pro-inflammatory cytokines and decreasing sIL-2R levels in treated samples. *uMakhonya®* also decreased the amount of secreted NO in PG-*S. aureus*-stimulated PBMCs. There is an urgent need for further studies on the mechanisms of action of *uMakhonya®* and other similar products being sold in supermarkets and pharmacies in South Africa. Standards must be developed to determine the types of plants that can be used in medicinal products based on traditional knowledge. This is highlighted by the use of plants in *uMakhonya®* that are not indigenous to South Africa such as *C. crispus* and *U. tomentosa*, and the use of regulated substances such as menthol.

## Abbreviations

ANOVA: Analysis of variance
ATM: African traditional medicine
ATP: Adenosine triphosphate
EU: Endotoxin units
GC-MS: Gas chromatography–mass spectrometry
GMP: Good manufacturing practice
IFN-γ: Interferon gamma
LAL: Limulus amebocyte lysate
MCC: Medicines control council
NAPPI: National pharmaceutical product index
NED: *N*-1-napthylethylenediamine dihydrochloride
NMR: Nuclear magnetic resonance
NO: Nitric oxide
NO_2_^−^: Nitrite
PBMCs: Peripheral blood mononuclear cells
PBS: Phosphate-buffered saline
PG: Peptidoglycan
PHA: Phytohaemagglutinin
RLU: Relative light units
*S. aureus*: *Staphylococcus. Aureus*
SANBS: South African National Blood Service
sIL-2R: Soluble interleukin-2 receptor
TNF-α: Tumor necrosis factor alpha

## References

[1] Ndhlala AR, Stafford GI, Finnie JF, Van Staden J (2011). Commercial herbal preparations in KwaZulu-Natal, South Africa: the urban face of traditional medicine. SA J Bot.

[2] Mander M, Ntuli L, Diederichs N, Mavundla K, Harrison S, Bhana R, Ntuli A (2007). Economics of the traditional medicine trade in South Africa. South African health review.

[3] Gqaleni N, Ngcobo M, Parboosing R, Naidoo A (2012). In vitro testing of African traditional medicines for cytotoxic, immune modulatory and anti-HIV activities. Afr J Trad Compl Altern Med.

[4] Ndhlala AR, Finnie JF, Van Staden J (2010). In vitro antioxidant properties, HIV-1 reverse transcriptase and acetylcholinesterase inhibitory effects of traditional herbal preparations sold in South Africa. Molecules.

[5] Olivier DK. The ethnobotany and chemistry of South African tonic plants. PhD Thesis. Johannesburg: University of Johannesburg; 2012. Available: https://ujdigispace.uj.ac.za/handle/10210/8094. Accessed 14 Jan 2014.

[6] Licciardi PV, Underwood JR (2011). Plant-derived medicines: a novel class of immunological adjuvants. Int Immunopharmacol.

[7] Lee H-K (2011). Immunomodulation and traditional medicines: a reductionist or holistic approach? - editorial. J Ethnopharmacol.

[8] Alamgir M, Uddin SJ, Chattopadhyay D (2010). Recent advances on the ethnomedicinal plants as immunomodulatory agents. Ethnomedicine: a source of complementary therapeutics.

[9] Agarwal SS, Singh VK. Immunomodulators: a review of studies on Indian medicinal plants and synthetic plants. PINSA B65. 1999;3-4:179–204.

[10] Yeap SK, Rahman MBA, Alitheen NB, Ho WY, Omar AR, Beh BK (2011). Evaluation of immunomodulatory effect: selection of the correct targets for immunostimulation study. Am J Immunol.

[11] Tzianabos AO (2000). Polysaccharide Immunomodulators as therapeutic agents: structural aspects and biologic function. Clin Microbiol Rev.

[12] Ngcobo M, Gqaleni N, Ndlovu V, Serumula M, Sibiya N (2016). Immunomodulatory effects of *umakhonya*®: a South African commercial traditional immune booster. S Afr J Bot.

[13] Leung TF, Wong KY, Wong CK, Fung KP, Lam CWK, Fok TF (2007). In vitro and clinical immunomodulatory effects of a novel pentaherbs concoction for atopic dermatitis. Br J Dermatol.

[14] Borish LC, Steinke JW (2003). Cytokines and chemokines. J Allergy Clin Immunol.

[15] Spelman K, Burns JJ, Nichols D, Winters N, Ottersberg S, Tenborg M (2006). Modulation of cytokine expression by traditional medicines: a review of herbal immunomodulators. Altern Med Rev.

[16] Caruso C, Candore G, Cigna D, Colucci AT, Modica MA (1993). Biological significance of soluble IL-2 receptor. Mediators Inflamm.

[17] Siedler S, Zimmermann H, Weiskirchen R, Trautwein C, Tacke F (2012). Elevated circulating soluble interleukin-2 receptor in patients with chronic liver diseases is associated with non-classical monocytes. BMC Gastroenterol.

[18] Gooding R, Riches P, Dadian G, Moore J, Gore M (1995). Increased soluble interleukin-2 receptor concentration in plasma predicts a decreased cellular response to IL-2. Br J Cancer.

[19] Lee SKW, Wong CK, Poon PMK, Ip PSP, Che CT, Chung KP (2006). In vitro immunomodulatory activities of a newly concocted traditional Chinese medicine formula: VI-28. Phytother Res.

[20] Mukherjee PK, Nema NK, Bhadra S, Mukherjee D, Braga FC, Matsabisa MG (2014). Immunomodulatory leads from medicinal plants. Indian J Tradit Knowl.

[21] Schrijver IA, van Meurs M, Melief M-J, Ang CW, Buljevac D, Ravid R (2001). Bacterial peptidoglycan and immune reactivity in the central nervous system in multiple sclerosis. Brain.

[22] Sorbara MT, Philpott DJ (2011). Peptidoglycan: a critical activator of the mammalian immune system during infection and homeostasis. Immunol Rev.

[23] Busia K (2005). Medical provision in Africa- past and present. Phytother Res.

[24] Okai Y, Higashi-Okai K, Ishizaka S, Yamashita U (1997). Enhancing effect of polysaccharides from an edible brown alga, *Hijikia fusiforme* (Hijiki), on release of tumor necrosis factor-α from macrophages of endotoxin-nonresponder C3H/Hej mice. Cancer Lett.

[25] Liu D, Keesing JK, He P, Wang Z, Shi Y, Wang Y (2013). The world’s largest macroalgal bloom in the yellow Sea, China: formation and implications. Estuar Coast Shelf Sci.

[26] Senthilkumar K, Manivasagan M, Venkatesan J, Kim S-K (2013). Brown seaweed fucoidan: biological activity and apoptosis, growth signaling mechanism in cancer. Int J Bio Macromolecules.

[27] Holdt S, Kraan S (2011). Bioactive compounds in seaweed: functional food applications and legislation. J Appl Phycol.

[28] Necas J, Bartosikova L (2013). Carrageenan: a review. Vet Med.

[29] Wijesekara I, Pangestuti R, Kim SK (2011). Biological activities and potential health benefits of sulfated polysaccharides derived from marine algae. Carbohyd Polym.

[30] Hutchings A, Scott AH, Lewis G, Cunningham AB (1996). Zulu medicinal plants: an inventory.

[31] Bors M, Michalowicz J, Pilarski R, Sicińska P, Gulewicz K, Bukowska B (2012). Studies of biological properties of *Uncaria tomentosa* extracts on human blood mononuclear cells. J Ethnopharmacol.

[32] Sandoval M, Charbonnet RM, Okuhama NN, Roberts J, Krenova Z, Trentacosti AM (2000). Cat’s claw inhibits TNF-α production and scavenges free radicals: role in cytoprotection. Free Radic Biol Med.

[33] Sheng Y, Bryngelsson C, Pero RW (2000). Enhanced DNA repair, immune function and reduced toxicity of *C-MED-100*, a novel aqueous extract from *Uncaria tomentosa*. J Ethnopharmacol.

[34] Sandoval M, Okuhama NN, Zhang X-J, Condezo LA, Lao J, Angeles FM (2002). Anti-inflammatory and antioxidant activities of cat’s claw (*Uncaria tomentosa* and *Uncaria guianensis*) are independent of their alkaloid content. Phytomed.

[35] Gutierrez RMP, Mitchell S, Solis RV (2008). *Psidium guajava*: a review of its traditional uses, phytochemistry and pharmacology. J Ethnopharmacol.

[36] De Wet H, Nkwanyana MN, van Vuuren SF (2010). Medicinal plants used for the treatment of diarrhoea in northern Maputaland, KwaZulu-Natal province, South Africa. J Ethnopharmacol.

[37] Holetz FB, Pessini GL, Sanches NR, Cortez DA, Nakamura CV, Filho BP (2002). Screening of some plants used in the Brazilian folk medicine for the treatment of infectious diseases. Mem Inst Oswaldo Cruz, Rio de Janeiro.

[38] Gelfand M, Mavi S, Drummond RB, Ndemera B (1985). The traditional medical practitioner in Zimbabwe.

[39] Rabe T, Van Staden J (1997). Antibacterial activity of South African plants used for medicinal purposes. J Ethnopharmacol.

[40] Eccles R (1994). Menthol and related cooling compounds. J Pharm Pharmacol.

[41] Baibars M, Eng S, Shaheen K, Alraiyes AH, Alraies MC. Menthol toxicity: an unusual cause of coma. Case Rep Med. 2012;1–3. 10.1155/2012/187039 PMC3521632 23251165

[42] Kengatharan KM, De-Kimpe S, Robson C, Foster SJ, Thiemermann C (1998). Mechanism of gram-positive shock: identification of peptidoglycan and lipoteichoic acid moieties essential in the induction of nitric oxide synthase, shock, and multiple organ failure. J Exp Med.

[43] Li H, Förstermann U (2000). Nitric oxide in the pathogenesis of vascular disease. J Pathol.

[44] Jacob T, Ascher E, Vorsanger M, Hingorani A, Kallakuri S, Yorkovich W, Schuzter R (2005). Decreased production of nitric oxide by pperipheral blood mononuclear cells of patients with peripheral vascular disease. Vasc Endovascular Surg.

[45] Coleman JW (2001). Nitric oxide in immunity and inflammation. Int Immunopharmacol.

[46] Patil GV, Dass SK, Chandra R (2001). *Artemisia afra* and modern diseases. Pharmacogenomics Pharmacoproteomics.

[47] Jimenez-Escrig A, Jimenez-Jimenez I, Pulido R, Saura-Calixto F (2001). Antioxidant activity of fresh and processed edible seaweeds. J Sci Food Agric.

[48] Ogunlana OE, Ogunlana OO (2008). In vitro assessment of the free radical scavenging activity of *Psidium Guajava*. Res J Agric Biol Sci.

[49] Romero-Jimenez M, Campos-Sanchez J, Analla M, Munoz-Serrano A, Alonso-Moraga A (2005). Genotoxicity and anti-genotoxicity of some traditional medicinal herbs. Mutat Res.

[50] Clement-Kruzel S, Hwang S-A, Kruzel MC, Dasgupta A, Actor JK (2010). Immune modulation of macrophage pro-inflammatory response by goldenseal and *Astragalus* extracts. J Med Food.

